# They Do Their Best, but Their Best Isn’t Good Enough: How Social Housing Can Support Older Tenants Aging in Place

**DOI:** 10.1093/geroni/igaa057.075

**Published:** 2020-12-16

**Authors:** Christine Sheppard, Andrea Austen, Sander Hitzig

**Affiliations:** 1 Sunnybrook Research Institute, Toronto, Ontario, Canada; 2 City of Toronto, Toronto, Ontario, Canada; 3 Sunnybrook Hospital, North York, Ontario, Canada

## Abstract

Toronto Community Housing (TCH) is the second largest social housing landlord in North America, and is home to over 27,000 older adults, half of whom live in 83 “seniors-designated” buildings. There is inadequate and inconsistent delivery of services in these buildings, negatively impacting tenants’ ability to age in place. We conducted two half-day consultations with service providers (n=74) and tenants (n=100) to identify strategies to improve unit condition, promote stable tenancies (i.e., prevent evictions) and enhance access to health and support services for older adults living in TCH. Through facilitated discussion, participants identified their top two recommendations for each priority area and reflected on the strategies that were hardest and easiest to implement, as well as the ones that would have the most and least impact on quality of life for older tenants. Participants recognized the need for more education as a way to empower older tenants and reduce stigma associated with unit condition issues (e.g., pest problems) and arrears. More frequent touch points with tenants was also recommended as a way to identify older adults at-risk of eviction and work proactively (instead of reactively) to support them. Service providers and tenants believed that system navigators working directly in the buildings would be a key facilitator to building trust and helping older tenants access needed services. Outcomes of the have several program and policy implications for TCH, as they partner with the City of Toronto to design a new integrated service model for the seniors-designated buildings.

